# Parents and Health Care Providers' Perspectives on Vital Signs Monitoring Technologies in the Neonatal Intensive Care Unit: An International Survey

**DOI:** 10.1055/a-2604-8329

**Published:** 2025-05-29

**Authors:** Eva Senechal, Daniel Radeschi, Robert Kearney, Wissam Shalish, Guilherme Sant'Anna

**Affiliations:** 1 Department of Experimental Medicine Department, McGill University, Montreal, Canada; 2 Department of Biomedical Engineering Department, McGill University, Montreal, Canada; 3 Neonatal Division, Department of Pediatrics, Montreal Children's Hospital, Montreal, Canada

**Keywords:** patient monitoring, wireless technology, survey, parents, health care providers

## Abstract

**Objective:**

This study aimed to assess the views of parents and neonatal intensive care unit (NICU) health care providers (HCPs) on current wired vital signs monitoring and future wireless alternatives.

**Study Design:**

Prospective cross-sectional survey was conducted between March and July 2023, targeting three groups: (1) NICU parents, (2) physicians, and (3) nurses and respiratory therapists (RT) and physiotherapists (PT). A 17-question survey was developed to assess several perspectives with current vital signs monitoring and a possible wireless monitoring system. NICU parents completed paper surveys and HCPs participated via an anonymous electronic survey. The original English survey was tailored for different respondent groups, translated into French, Spanish, and Portuguese, and distributed through neonatal research networks. Responses from each group were analyzed as totals (%), with within-group comparisons assessed using the Wilcoxon signed-rank test. Additionally, between-group comparisons were conducted using the chi-square test of independence or Fisher's exact test, as appropriate.

**Results:**

A total of 1,141 responses were included (25 parents, 438 physicians, and 678 nurses, RTs, and PTs). Only 52% of parents were satisfied with current wired systems; 68% reported wires hindered infant handling, and 52% cited interference with skin-to-skin care. Both physicians and HCPs expressed low satisfaction with the current system. Common concerns included tangling, skin irritation, and workload. Support for wireless technology introduction was high across all groups (parents = 60%, physicians = 91%, and nurses, RTs, and PTs = 87%), with main perceived benefits including improved kangaroo mother care (KMC), reduced patient discomfort, and enhanced bonding. All groups expressed accuracy, safety, battery life, and cost concerns of a possible wireless system.

**Conclusion:**

Parents and HCPs are generally dissatisfied with the current NICU vital signs monitoring systems, primarily due to concerns with wires and cables and interference with KMC. Wireless technologies were mostly supported, but data on reliability, safety, and economic feasibility will be critical for development and successful implementation.

**Key Points:**

- Parents and HCPs dislike wired systems due to tangling, skin irritation, and interference with care.
- Support for wireless monitoring was viewed positively by parents and very positively by HCP.
- Wireless systems were seen as beneficial for KC, reducing patient discomfort, and improving bonding.
- However, each group expressed concerns about a potential future wireless monitoring system.
- Accuracy, battery life, radiation, and cost must be addressed before wireless systems can be adopted.

Approximately 10 to 12% of all newborns are admitted to the neonatal intensive care unit (NICU) after birth. 1 Clinical care of these patients heavily relies on continuous monitoring of electrocardiogram (ECG) and heart rate (HR), oxygen saturation (SpO _2_ ), respiratory rate (RR), and axillary temperature (T _ax_ ). 2 Information from these vital signs is used for assessment of clinical states and management decisions. 2

Current technology for vital signs monitoring includes skin sensors connected to large and expensive bedside monitors by wires and cables. In most patients, raw signals and average values of HR, SpO _2_ , RR, and T _ax_ are continuously displayed. However, this monitoring system poses some challenges for patients, parents, and health care professionals (HCP) as the wires can tangle around the newborn body, restrict the patient's movement, and cause discomfort or pressure sores. For that, regular care involves frequent removal, reapplication, and readjustments of the sensors, which may harm the fragile neonatal skin, cause pain, and/or interrupt resting or sleeping. For parents, the presence of multiple wires and cables can cause intimidation and additional stress, acting as a barrier to skin-to-skin contact for fear of disconnecting the sensors or wires, and interfering with the regular monitoring. 3 4 5 Moreover, it may increase HCPs workload as wires and cables may touch contaminated surfaces or become soiled with urine, blood, or stools, increasing the risks of nosocomial infections. 3 6 7 Hence, nurses must constantly inspect, sanitize, reposition, or replace components of this system. 8

Despite these limitations, NICU monitoring has remained largely unchanged since the 1980s but recent advancements in wireless and wearable sensor technology for health care have spurred efforts to develop wireless monitoring systems for hospitalized patients, including NICU patients. 9 10 11 12 13 14 Nevertheless, validation and implementation of new technologies in clinical settings is a complex process that must establish its safety, feasibility, and accuracy. Moreover, it should also address the perspectives and concerns of users. Unfortunately, existing research including parents and HCPs was small and not specifically focused on their perspectives on existing or emerging monitoring technologies and their potential use in the NICU. 3 As part of a comprehensive investigation of wireless sensors in the NICU, parents, and HCPs' views on the current wired standard of care and the possible development and implementation of a wireless monitoring system were assessed using an international survey.

## Materials and Methods

This study was conducted between March and July 2023 with the use of an e-survey (LimeSurvey). Exemption for informed consent was obtained from the Institution Research Ethics Board, as the responses and their aggregate analysis were considered secondary use of anonymous information (Tri Council Policy Statement 2022, Article 2.4).

## Survey Development

A survey was developed with input from neonatologists, nurses, and parents at the Montreal Children's Hospital (MCH) NICU (see Supplementary Material S1 , available in the online version only). The survey items were selected based on observations made in the course of developing and conducting a prior research study examining the feasibility, safety, and accuracy of a specific wireless monitoring system in the NICU (NCT04956354). 15 Prior knowledge was based on focus groups with parents conducted as part of the development of this study, and a short survey of parents and nurses who participated in the study. A preliminary survey was developed and circulated to parents, neonatologists, and nurses at the MCH NICU. This process was re-iterated until all groups were satisfied with the contents and phrasing of the survey. The final 17-question survey included multiple choice questions, Likert scales, and short-answer responses and had three main sections: (1) general information (relationship to the patient, gestational age (GA) at birth, duration of NICU stay, profession, and years of experience), (2) experience with the current monitoring system, and (3) perspectives on a possible wireless monitoring system for NICU patients. The order of the questions was not randomized.

The respondent groups were selected to capture the perspectives of key NICU stakeholders who directly engage with the bedside monitoring system, ensuring that our insights reflect their diverse experiences. To ensure relevance for different respondent groups, the survey was also tailored for three specific groups: (1) parents, (2) physicians, and (3) nurses, physiotherapists (PTs), and respiratory therapists (RTs). For the parent survey, lay language was used to enhance understanding. In the HCP surveys, the phrasing and contents of the base survey were modified to address the specific roles and expertise of the respondents. The revised surveys were re-circulated to a small group of neonatologists and nurses at the MCH NICU for additional feedback. The final parent survey was translated into French, while the HCP surveys were translated into French, Spanish, and Portuguese to allow broader accessibility. Translations were completed by native-speaking neonatologists for each language included in the survey to ensure the contents and terminology of the survey were appropriate. Participation in the survey was entirely voluntary. The online surveys were designed and reported in accordance with the Checklist for Reporting Results of Internet E-Surveys (CHERRIES; see Supplementary Material S2 , available in the online version only). 16

## Distribution

### Parents

The survey was distributed to parents of infants admitted to the NICU for a minimum of 5 days to ensure familiarization with the environment and equipment. Eligible parents received a paper copy of the survey with no time restriction for completion. Survey copies were collected and transcribed into an electronic format using Excel. A convenience sample size of 25 parents was determined based on the number of eligible parents willing to complete the survey during the study period. No incentives were provided.

### Health Care Professionals

Links to the surveys in each language were distributed via email and social media platforms by the principal investigators (GS and WS) to publicly listed NICU directors, professional neonatal research networks of global neonatal practitioners, and unit directors. The survey was open, allowing NICU directors to share the link with their staff. Participants could navigate forward and backward within the three-page survey to modify the answers. There was no time limit for completing the survey, but the duration of survey completion was recorded. The survey could be accessed and completed on a tablet, phone, or computer. Data collected included demographics, survey language, professional title, and years of practice. No identifying information, such as names or IP addresses, was collected to protect participant privacy. Timestamp information was collected, and results were exported as CSV files for analysis. No incentives were provided for completing the survey. A convenience sample of 1,000 responses was planned.

### Analysis

Survey data were imported into MATLAB for analysis. Incomplete responses were included if sufficient basic demographic information was available to confirm the respondent's eligibility. Descriptive and statistical analyses were conducted for all questions, with results reported as numbers and percentages for each group. Additionally, to assess differences in attitudes toward wired versus wireless monitoring, responses were converted to ordinal values (1 = very dissatisfied/ very negative, 2 = dissatisfied/negative, 3 = neither satisfied nor dissatisfied/neither positive nor negative, 4 = satisfied/positive, to 5 = very satisfied/very positive) and analyzed using the Wilcoxon signed-rank test. Attitudes ranked 1 to 2 were considered negative, 3 was considered neutral, and 4 to 5 were considered positive. The Likert scale ordinal value conversions for each question are specified in Supplementary Table S1 (available in the online version only). A *p* -value <0.05 was considered statistically significant.

Additional analyses explored intragroup differences. Among parents, the influence of infant GA and NICU stay duration on responses was examined by converting Likert scale responses to integers and conducting correlational analysis. For health care providers (HCPs), a chi-squared test of independence to assess whether years of practice, categorized in the survey, influenced responses.

Intergroup differences among HCP subgroups (physicians vs. nurses, RT, and PT) were analyzed separately using a chi-squared test of independence, and a *p* -value <0.05 was considered statistically significant. Due to the large differences in sample sizes and sample composition (single center vs. multicenter), statistical comparisons between HCP and parent responses were considered inappropriate.

## Results

**Fig. 1:**
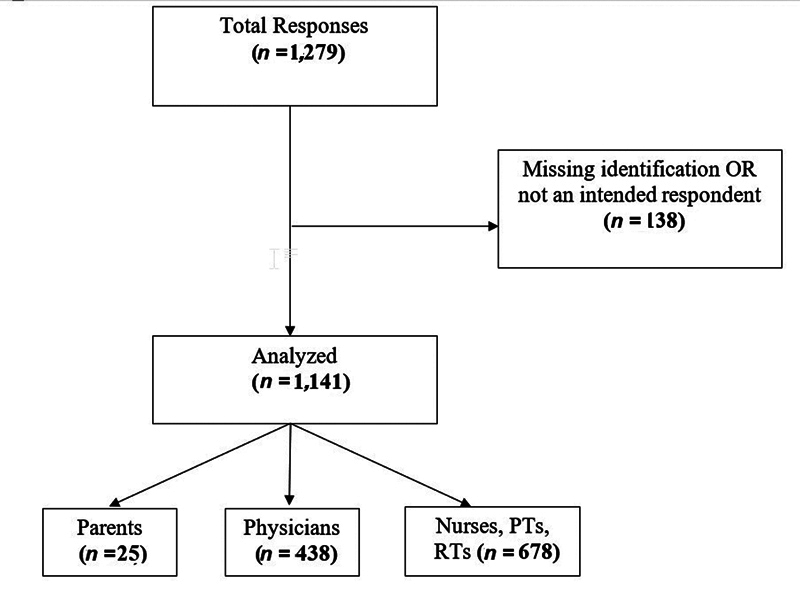
Flowchart. PT, physiotherapists; RT, respiratory therapists.

A total of 1,279 responses were obtained: parents = 25, and HCPs = 1,254 (physicians = 525 and nurses, RTs, and PTs = 729); 138 surveys were not included due to lack of identification, or because respondents were not from the groups of interest (e.g., nursing student and medical student). Surveys with partial responses were accepted. Thus, a total of 1,141 responses were included: parents = 25, HCPs = 1,116; physicians =438, and nurses, RTs, and PTs = 678 ( Fig. 1 ). The majority of parent surveys were completed by mothers ( Table 1 ).

**Table 1:** Demographic information regarding identity in relation to NICU patients and length of experience in the NICU

Parents a	*n* = 25
Mother	21 (81)
Father	5 (19)
GA of infant	30 (7–33)
Number of days in the NICU	21 (8–28)
Physicians	*n* = 438
Title	
Neonatologist	376 (86)
Neonatal fellow	24 (5)
Pediatrician	21 (5)
Pediatric resident	12 (3)
Physician	5 (1)
Work experience (y)	
<2	26 (6)
2–5	65 (15)
6–10	57 (13)
>10	290 (66)
Nurses, RTs, and PT's	*n* = 678
Title	
Registered nurse	488 (72)
PT	139 (21)
Neonatal nurse practitioner	48 (7)
RT	3 (<1)
Work experience (y)	
<2	66 (10)
2–5	120 (18)
>5–10	141 (21)
>10	350 (52)
No answer	1 (<1)

Abbreviations: NICU, neonatal intensive care unit; PT, physiotherapist; RT, respiratory therapist.

Note: Results are presented as *n* (%) or median (IQR).

a One set of parents indicated that both mother and father completed the survey together.

### Parents

**Fig. 2:**
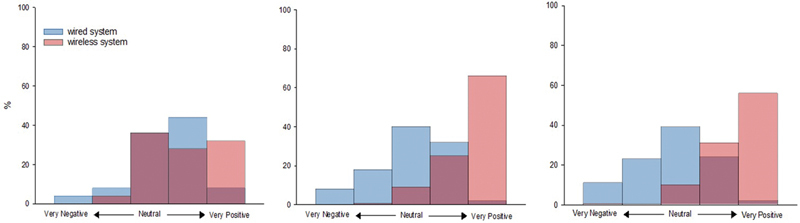
Attitudes toward a wired and a future wireless monitoring system in the NICU. Satisfaction levels were classified as very dissatisfied, dissatisfied, neither satisfied nor dissatisfied, satisfied, and very satisfied for the wired system (Q1). Perceptions of wireless systems were classified as very negative, negative, neither positive nor negative, positive, and very positive (Q6 and Q10). Red = current monitoring system and blue = future wireless technology in the NICU and the darker color indicates the overlap of the red and blue bars. Response from parents ( **A** ), physicians ( **B** ), and nurses, respiratory therapists (RTs), and physiotherapists (PTs). A *p* -value <0.05 was considered statistically significant. The 95% confidence intervals for all percentages presented in panels ( **A–C** ) are provided in Supplementary Table S2 (available in the online version only).

Current wired monitoring system: Thirteen parents (52%) reported as satisfied, but a significant proportion (68%) expressed concerns about the wires acting as a barrier to infant handling and 14 (58%) indicated that the current system interfered with skin-to-skin care ( Fig. 2A ; Supplementary Table S2 , available in the online version only). The 95% confidence intervals (CI) for the percentages for the perceived interference with skin-to-skin care presented in Fig. 2A are provided in Supplementary Table S2 (available in the online version only). Other commonly reported issues included tangling of wires around the infant's body (52%), and frequent disconnection of wires (44%) and cables (48%). A detailed description of all responses is presented in Table 2 .

**Table 2:** Perceived impact of the wired standard bedside monitoring system among NICU parents, physicians, and nurses, PT, and RT

Survey item	Parents	Physicians	Nurse, PT, and RT
Main concerns	*n* = 25	*n* = 380	*n* = 593
Inaccurate readings	5 (20)	219 (58)	352 (59)
False alarms	14 (56)	226 (59)	428 (72)
Effect of sensor adhesives	11 (44)	294 (77)	405 (68)
Too many sensors applied to the skin	14 (56)	254 (67)	421 (71)
Too many wires around the infant	12 (48)	283 (75)	428 (72)
Barriers to handling the infant because of the multiple wires and cables	17 (68)	229 (60)	378 (64)
Issues encountered	*n* = 25	*n* = 380	*n* = 593
Skin lesions associated with ECG adhesives	9 (36)	253 (67)	371 (63)
Skin lesions associated with oxygen saturation sensor	3 (12)	247 (65)	348 (59)
Pressure sores associated with ECG adhesives	4 (16)	86 (23)	116 (20)
Pressure sores associated with oximeter sensors	4 (16)	193 (51)	252 (43)
Parents afraid to handle infants because of the wires and cables	18 (72)	275 (72)	513 (87)
Wires tangled around the infant's chest or limbs	13 (52)	248 (65)	440 (74)
Wires soiled—requiring replacement or cleaning	5 (20)	148 (39)	354 (60)
Cables soiled—requiring replacement or cleaning	5 (20)	135 (36)	269 (45)
Wires disconnected	11 (44)	221 (58)	327 (55)
Cables disconnected	12 (48)	171 (45)	194 (33)
Wires broken or not working—requiring replacement	4 (16)	208 (55)	367 (62)
Cables are broken or not working—requiring replacement	1 (4)	177 (47)	330 (56)
Interferes with parents' bonding with their infant	*n* = 25	*n* = 376	*n* = 587
Strongly disagree	3 (12)	11 (3)	18 (3)
Disagree	6 (24)	61 (16)	104 (18)
Neither agree nor disagree	10 (40)	70 (19)	146 (25)
Agree	4 (16)	193 (51)	267 (45)
Strongly agree	2 (8)	41 (11)	52 (9)
Proportion of a nurse's shift time is spent taking care of sensors/wires/cables (%)			*n* = 586
<5			198 (34)
5–10			213 (36)
11–20			88 (15)
21–30			48 (8)
31–40			28 (5)
>40			11 (2)
Frequency of sensors/wires replacement during a 12-h shift			*n* = 588
Never			69 (12)
Once			218 (37)
Twice			37 (6)
>2 times			81 (14)
I don't know			52 (9)
Other			131 (22)
During a regular shift (12 h) number of times the nurse addresses issues related to wires			*n* = 585
Median (IQR)			5 (3–6)
How often are the cables replaced during a 12-h shift?			*n* = 588
Not at all			309 (53)
Once			97 (17)
Twice			26 (4)
>2 times			25 (4)
I don't know			68 (12)
Other			63 (11)

Abbreviations: PT, physiotherapist; RT, respiratory therapist.

Note: Partially completed surveys were included in this analysis, therefore the number of respondents for each survey question are presented, and responses are presented as *n* (%) or median (IQR).

Wireless monitoring technology: Fifteen parents (60%) expressed positive attitudes toward a potential introduction of wireless monitoring in the NICU ( Fig. 2A ; Supplementary Table S3 , available in the online version only). The 95% CI for the percentages of attitudes toward introducing wireless technology presented in Fig. 2A are provided in Supplementary Table S3 (available in the online version only). Perceived benefits included better kangaroo mother care (KMC) experience (96%), increased KMC duration (76%), reduced patient discomfort (68%), and improved sleep quality and duration (52%). Twelve parents (48%) reported concerns regarding safety and accuracy. The most frequently cited concerns included data quality and reliability of the vital signs taken with the wireless technology (68%) and the need for a “user-friendly” system (52%). Additionally, most parents believed wireless technology would be more expensive (72%) and expressed worry about the potential radiation levels associated with Bluetooth technologies (80%). A detailed description of parent perspectives on a wireless monitoring system for the NICU is documented in Table 3 .

**Table 3:** Perceived impact of a future wireless bedside monitoring system among NICU parents, physicians, and nurses, PT, and RT

Survey item	Parents	Physicians	Nurse, PT, and RT
Concerns about safety and accuracy	*n* = 25	*n* = 357	*n* = 568
Strongly disagree	4 (16)	25 (7)	32 (6)
Disagree	2 (8)	78 (22)	117 (21)
Neither agree nor disagree	7 (28)	83 (23)	139 (24)
Agree	12 (48)	145 (41)	226 (40)
Strongly agree	0 (0)	26 (7)	34 (6)
Main concerns about implementation	*n* = 25	*n* = 360	*n* = 575
Accuracy (good and reliable data)	17 (68)	284 (79)	577 (83)
Challenges related to “user-friendliness” of technology	14 (56)	155 (43)	187 (33)
Safety of the wireless technology	8 (32)	210 (58)	308 (54)
Size of the wireless sensors	9 (36)	144 (40)	251 (44)
Weight of the sensors	8 (32)	127 (35)	240 (42)
Battery life of the wireless technology	9 (36)	198 (55)	341 (59)
Other	3 (12)	36 (10)	44 (8)
Cost perception	*n* = 25	*n* = 360	*n* = 567
Less expensive than the current wired system	1 (4)	19 (5)	27 (5)
More expensive than the current wired system	18 (72)	247 (69)	411 (72)
No idea	6 (24)	81 (23)	126 (22)
Other	0 (0)	10 (3)	3 (0.5)
Changes on outcomes	*n* = 25	*n* = 360	*n* = 568
Improve kangaroo care (KC) experience	24 (96)	310 (86)	520 (92)
Increase the amount of time in KC	19 (76)	223 (62)	326 (57)
Reduce patient discomfort	17 (68)	284 (79)	425 (75)
Decrease patient pain	9 (36)	176 (49)	262 (46)
Improve sleep	13 (52)	202 (56)	324 (57)
Improve weight gain	5 (20)	90 (25)	166 (29)
Earlier discharge home	4 (16)	75 (21)	138 (24)
Lower hospital resources and costs	–	107 (30)	176 (31)
Impact on the physical interaction between parents and infants	*n* = 25	*n* = 357	*n* = 568
Very negative	0 (0)	0 (0)	0 (0)
Negative	0 (0)	0 (0)	3 (0.5)
Neutral	1 (4)	20 (6)	28 (5)
Positive	14 (56)	106 (30)	177 (31)
Very positive	10 (40)	231 (65)	360 (63)
Concerns with radiation levels	*n* = 24	*n* = 357	*n* = 568
Not at all worried	5 (21)	164 (46)	274 (48)
Somewhat worried	16 (67)	178 (50)	261 (46)
Very worried	3 (13)	15 (4)	33 (6)
Add comments	*n* = 6	*n* = 37	*n* = 79

Abbreviations: PT, physiotherapist; RT, respiratory therapist.

Note: Results are presented as *n* (%), or median (IQR). Partially completed surveys were included, therefore the number of respondents for each survey question is presented.

### Physicians

**Fig. 3:**
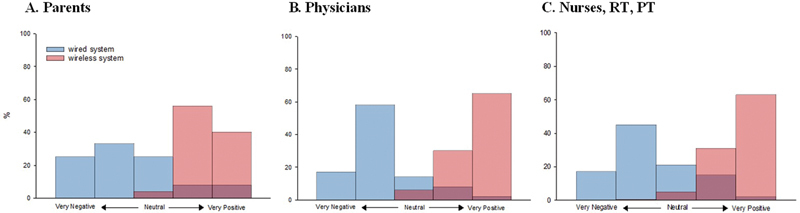
Impact of wired versus wireless monitoring on parent–infant contact in the NICU. Views on the current wired system impact are based on responses to strongly disagree, disagree, neither agree nor disagree, agree, and strongly agree on the statement that the wired monitoring system interferes with KC (Q3). Responses for the impact of the wireless system on KC range: very negative, negative, neither negative nor positive, positive, very positive (Q11 and Q14). Red = current monitoring system and blue = future wireless technology in the NICU, and the darker color indicates the overlap of the red and blue bars. Response from parents (panel **A** ), physicians (panel **B** ), and nurses, respiratory therapists (RTs), and physiotherapists (PTs). A *p* -value <0.05 was considered statistically significant. The 95% confidence intervals for all percentages presented in panels ( **A–C** ) are provided in Supplementary Tables S2 and S3 (available in the online version only).

The majority of physicians were neonatologists (86%) with over 10 years of practice (66%; Table 1 ). Only 34% reported they were satisfied with the current monitoring system ( Fig. 2B ; Supplementary Table S2 , available in the online version only). The 95% CI for the satisfaction percentages presented in Fig. 2B are provided in Supplementary Table S2 (available in the online version only). Primary concerns included issues related to skin sensor adhesives (77%), the number of sensors applied to the skin (67%), and the number of wires surrounding the infant (75%). Physicians identified parental fear of holding the infant (72%), wires tangling (65%), and skin lesions caused by ECG adhesives (67%) or SpO _2_ sensors (65%) as the most common problems of the current system. Additionally, most physicians agreed that the current system interferes with parent–infant skin-to-skin contact (75%; Fig. 3B ; Supplementary Table S2 , available in the online version only) and bonding (62%). The 95% CI for the percentages representing interference with skin-to-skin contact in Fig. 3B are provided in Supplementary Table S2 (available in the online version only). A detailed description of physicians' perspectives on the current monitoring system is documented in Table 2 .

Nearly all physicians (91%) expressed positive attitudes toward the potential introduction of wireless monitoring technology in the NICU ( Fig. 2B ; Supplementary Table S3 , available in the online version only). The 95% CI for the percentages of attitudes toward the introduction of wireless monitoring are presented in Fig. 2B and are provided in Supplementary Table S3 (available in the online version only). The perceived benefits included improvements in the KMC experience (86%) and duration (62%), and reduced patient discomfort (79%; Table 3 ). Most physicians believed wireless technology would have a highly positive impact on encouraging physical interaction between parents and infants (65%). However, 48% expressed concerns regarding the safety and accuracy of wireless technology in the NICU. The primary concerns included accuracy (79%), safety (58%), battery duration (55%), and radiation levels (54%). Additionally, 69% of physicians believed that wireless systems would be more expensive than the current systems. A detailed description of physicians' perspectives on a wireless monitoring system in the NICU is documented in Table 3 .

Statistical analysis revealed no systematic differences in physician responses based on years of experience, except for one question. Specifically, while most physicians with less than 2 years (70%), 2 to 5 years (73%), and over 10 years (80%) of experience agreed that the current system interferes with KMC, only 57% of those with 5 to 10 years of experience expressed agreement. Detailed results are available in Supplementary Table S4 (available in the online version only).

### Nurses, Respiratory Therapists, and Physiotherapists

Respondents were primarily nurses (72%), with over 10 years of experience (52%). Only a minority (33%) reported satisfaction with the current monitoring system ( Fig. 2C ; Supplementary Table S2 , available in the online version only). The 95% confidence satisfaction percentages presented in Fig. 2C are provided in Supplementary Table S2 (available in the online version only). Key concerns included false alarms (72%), the number of wires around the infant (72%), and number of sensors applied to the skin (71%). The most commonly reported issues were parental fear of holding the infant (87%), wires tangling (74%), skin lesions caused by ECG adhesives (63%), and broken wires (62%). Most respondents felt that the current monitoring system interfered with parents' ability to perform skin-to-skin care (71%; Fig. 3C ; Supplementary Table S2 , available in the online version only), while just over half believed it hindered parent–infant bonding (54%; Table 2 ). The 95% confidence percentages for interreference with skin-to-skin care are presented in Fig. 3C and are provided in Supplementary Table S2 (available in the online version only).

The majority of respondents estimated that nurses spend less than 10% of their shift managing sensors, wires, and cables (70%; Table 2 ). During a 12-hour shift, nurses estimated that they needed to fix issues with wires and cables five times per day (IQR: 3–6/day). The most commonly reported frequency of changing sensors and wires during a 12-hour shift was once (37%; Table 2 ), and 53% reported no need to change cables ( Table 2 )

The introduction of wireless monitoring technology in the NICU was viewed positively by a large majority of nurses, PTs, and RTs (87%). The primary perceived benefits included improved KMC experience (92%), reduced patient discomfort (75%), increased KMC duration (57%), and improved sleep (57%). Most respondents believed a wireless system would positively impact physical interaction between parents and infants (63%; Fig. 3C ; Supplementary Table S3 , available in the online version only). The 95% CI for the percentages of perceived impact on physical interaction presented in Fig. 3C are provided in Supplementary Table S3 (available in the online version only). However, nearly half of the respondents (46%) expressed concerns about safety and accuracy. Primary concerns were data accuracy and reliability (83%), safety (54%), battery duration (59%), and radiation levels (52%). Additionally, 72% of respondents anticipated that wireless systems would be more expensive than current wired systems ( Table 3 ).

Statistical analysis was conducted to assess intergroup differences based on years of practice and answers, but no significant associations were found. Detailed results are provided in Supplementary Table S5 (available in the online version only).

### Physicians versus Nurses, RTs, and PTs

For most questionnaire items, there were no significant differences between nurses, RT, PT, and physician responses. Nurses, RTs, and PTs expressed greater dissatisfaction with the current monitoring system compared with physicians. Conversely, physicians perceived that the current wired monitoring system had a more pronounced negative impact on skin-to-skin contact and parent–infant bonding. Detailed results for all statistical comparisons between these groups are provided in Supplementary Table S6 (available in the online version only).

## Discussion

**Fig. 4:**
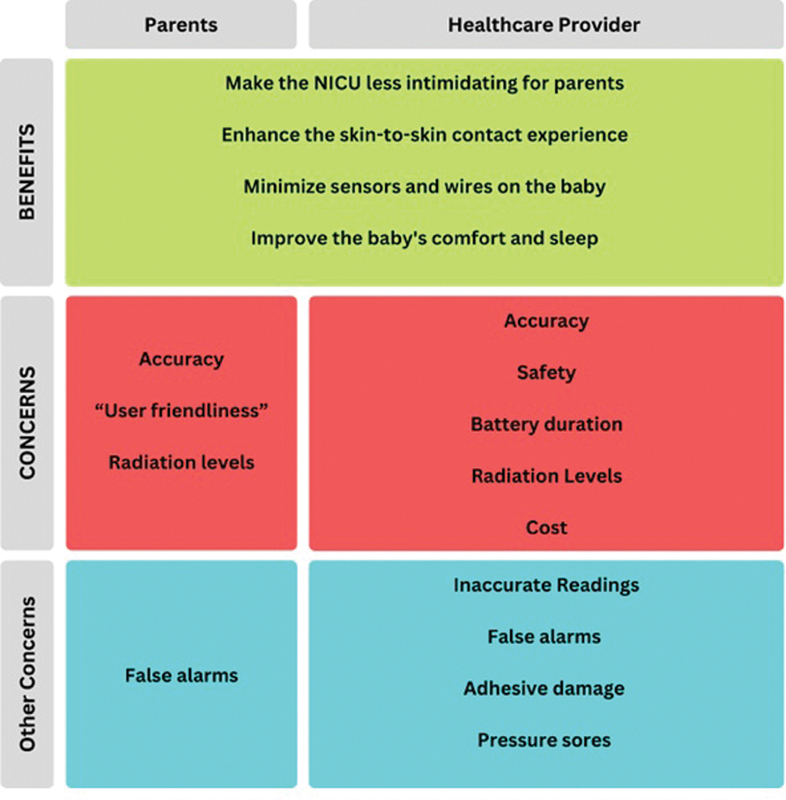
Summary of the perspectives toward the introduction of wireless technology in the NICU.

The current study provides one of the largest surveys to date on parents' and HCPs' perceptions on the specific topic of vital signs monitoring systems in the NICU. Our results showed a widespread dissatisfaction with the current monitoring technology and a generally positive, though cautious, enthusiasm for implementing wireless monitoring technology in the NICU ( Fig. 4 ).

### Parents

Current understanding of parents' perceptions of NICU monitoring systems remains limited. Studies examining the impact of NICU admission on parents have occasionally touched on this topic and consistently showed significant parental stress and trauma, often exacerbated by the highly technical and medicalized environment, including the presence of wires and cables. 17 18 19 20 21 22 23 Notably, three studies have reported on the impact of monitoring technologies on NICU parents. Semi-structured interviews were conducted with seven NICU parents who reported feeling deeply overwhelmed by the overall highly technical environment. 21 Similarly, a large survey including 248 NICU parents explored their perceptions of various medical technology products (MTPs), including vital signs monitoring. 19 MTPs were perceived as significant obstacles to parents' ability to see, touch, and participate in their infant's care. 19 To date, only one published study has specifically examined the effect of vital sign monitoring wires on KMC, using semi-structured interviews with six NICU parents. Nearly all parents identified the wires as overwhelming and a barrier to KMC. 3 At the MCH, upon arriving at the NICU parents are provided with information/education about KMC and encouraged to engage in this practice. Internal data shows the average time between admission and initiation of KMC is 3.5 days and 90% of infants receive KMC on most days of the week (40% daily and 50% on most days). Ideally, when children are eligible, KMC should be practiced on a daily basis, and obstacles to achieving this goal for all patients should be addressed. Importantly, 72% of parents in our survey stated they were afraid to handle their infant because of wires and cables, and 58% stated they felt it was an obstacle to KMC. Additionally, our survey revealed other aspects of parental dissatisfaction, including concerns about tangling of the wires, disconnection, or malfunctioning of the sensors.

Currently, only three studies have directly addressed parental attitudes toward wireless monitoring systems. 3 24 25 Bonner et al, also explored perceptions of introducing a wireless system and all parents responded positively citing primary benefits comparable to our results. These included reduced parental anxiety, improved physical interaction, and enhanced infant comfort. 3 Similar to our findings, parents expressed concerns about signal reliability and quality, battery duration, and sensor size. Ginsburg et al interviewed 10 parents of a teaching hospital in Kenya regarding their views on a new wireless monitoring sensor. 24 Sixty percent of parents reported the wireless sensor more comfortable and 70% stated the technology was very easy to use. Notably, 80% reported concerns about the reliability and safety of wireless transmission, and nearly one-third expressed concerns about the size. Peterson et al surveyed 51 parents of infants born at a maternity hospital in the United Kingdom about their experience with a new wireless monitoring system placed on the infant's head. 25 The majority found the device useful, comfortable, and easy to fit. Fifty percent reported it was comfortable to use during KMC, but multiple respondents expressed concerns about the device's appearance and stability. Our findings largely align with these studies, demonstrating parents' enthusiasm for wireless technologies, particularly their impact on parental experience, KMC, and infant comfort. Bluetooth emits nonionizing radiation at low power levels, which is considered insufficient to damage DNA and cause cancer. Also, research on Bluetooth radiation has been reviewed and considered to be safe by the FDA and WHO 26 Nevertheless, parents expressed apprehension about radiation exposure. To our knowledge, this is the first study to include parents' concerns about this issue, underscoring the need for further research to fully understand potential long-term impacts.

### Health Care Professionals

There is also limited research on HCPs' perceptions of NICU monitoring systems, with most existing data primarily focusing on alarm fatigue and accuracy. 3 27 28 29 Bonner et al, applied semi-structured interviews with seven NICU nurses that reported a positive view of the current monitoring system. 3 However, despite this concerns related to increased handling, positioning challenges, clutter and tangling of wires, adhesive-related issues, and risk of pressure sores were highlighted. Two additional studies, conducted by the same research group, examined the perspectives of ICU physicians, nurses, and RT regarding current monitoring systems. 27 28 The first study involved semi-structured interviews with five physicians, six nurses, and four RT, in which participants frequently expressed concerns about false alarms and wire entanglement. 27 In a subsequent larger survey involving 62 ICU nurses and 24 ICU physicians, only 42% of respondents reported satisfaction with the current system. 28 Additionally, only 51% believed the system guaranteed a high level of patient safety, with concerns regarding false alarms and an excessive number of sensors and cables around the patient. 28 HCPs in our survey expressed similar frustrations with NICU patients, particularly regarding the impact on patient discomfort and pressure sores.

These three studies also revealed a generally positive attitude toward wireless monitoring technology. Bonner et al reported almost all nurses had a positive attitude toward the introduction of wireless sensors, citing benefits such as improved comfort, reduced parental stress, and enhanced KMC. 3 However, they also expressed concerns regarding the reliability, size, weight, and battery life of the sensors. In a small qualitative study, HCPs emphasized the importance of a wireless, noninvasive, interoperable, and intuitive system that could reduce false alarms. 27 Importantly, they expressed concerns about trusting novel technologies, potential loss of clinical skills, increased workload, and lack of understanding of the technologies. 27 In the larger follow-up survey, 93% of staff supported the introduction of wireless sensors, reiterating a desire for improvements in reducing false alarms. 28 Furthermore, most participants (69%) expressed trust in novel technologies. In our current survey, nearly all HCPs supported the introduction of wireless systems and also expressed apprehension regarding the reliability and accuracy of wireless systems and potential safety risks related to sensor size, weight, and radiation exposure. Concerns about the increased cost of wireless technology also emerged. Interestingly, existing research on novel wireless technologies in the NICU often provides little to no analysis of costs associated with the development and implementation of these technologies despite their importance for widespread adoption. 10 30 31 32 The lack of economic feasibility studies represents a major gap that should be addressed.

## Limitations

The study has some limitations. We used social media and emails to distribute the survey and there is always an inherent bias toward a certain type of respondent and multiple participants from a limited number of centers. Also, it was not possible to identify or prevent multiple responses from the same individual and to ascertain the total number of participants. However, web-based tools have been increasingly used to maintain or improve communication and the speed of interaction between and across different stakeholders in health care. 33 Indeed, articles indexed on PubMed involving social media have practically doubled each year for the past 10 years, with ongoing discussions on how social media may facilitate collaborations and knowledge-sharing. 34 HCPs responses were intentionally solicited from individuals and may not reflect a NICU practice. However, the current survey was simple and pragmatic, which helped us acquire more than 1,000 answers from different healthcare professionals, quickly and with no associated costs. Additionally, while this survey was translated into multiple languages to capture responses from diverse NICU environments, it was not translated into some widely spoken languages, such as Mandarin and Hindi. This limitation may reduce the generalizability of the findings to NICUs in some geographic locations. Furthermore, since the survey was distributed through international neonatology networks, the responses are likely biased toward centers that are more actively engaged in global research collaborations, potentially underrepresenting perspectives from institutions with limited international connectivity such as rural centers. In terms of the insights obtained from parents, the generalizability of these findings may be more limited than those obtained from HCP providers as only parents from one NICU were included in the survey. This was because our goal was to assess the perspective of parents currently in the NICU, and the practical challenges of accessing parents from NICU's external to our institution. The decision to combine perspectives from the smaller MCH parent survey and the larger international survey was made to ensure that the views of key NICU stakeholders, who directly experience the NICU environment and modern technologies, were adequately represented in our investigation. Finally, this study does not utilize any qualitative data collection methods, such as focus group discussions, in-depth interviews, or key informant interviews. While the quantitative survey provided measurable insights, qualitative approaches could enrich the findings by capturing nuanced perspectives, contextual factors, and participant experiences that may not be fully reflected in structured survey responses.

## Conclusion

This study presents a large international cross-sectional survey of parents, physicians, nurses, and RTs and PTs on vital signs monitoring in the NICU. The majority of parents and HCPs are dissatisfied with the current monitoring systems, perceiving it as a significant barrier to KMC. A cautious optimism was expressed about the introduction of wireless technology, highlighting its potential benefits for KMC and parental experience, but also articulating concerns about accuracy and safety. Future research on wireless monitoring technology should prioritize evaluation of accuracy, radiation exposure, reliability, and economic feasibility of implementing these technologies in NICUs.
