# Supplementary material for: Parents and Health Care Providers' Perspectives on Vital Signs Monitoring Technologies in the Neonatal Intensive Care Unit: An International Survey
Source: Am J Perinatol. 2025 May 29;43(2):245–56. doi: 10.1055/a-2604-8329 (PMC12768579; doi:10.1055/a-2604-8329)
Supplement: Supplementary file 1 — Supplementary Material [file 10-1055-a-2604-8329-s25feb0103.pdf]

Supplementary Material S1 Survey

1A. Parent Survey

Dear Sir/Madam,

The Montreal Children's Hospital is doing a project to transform the way we care for our patients. This is called the Smart Hospital Project. The project will test new wireless technology that monitors vital signs, such as heart rate, oxygen levels, breathing rate, and temperature in the neonatal intensive care unit (NICU). Currently, the NICU monitors vital signs with wires that connect each baby to a monitor.

We want to know your opinion about the current monitors that use wires and the new monitors which will be wireless. Your input is important to ensure the Smart Hospital Project is a success. If you agree to participate, we will ask that you complete this survey about your experience in the NICU. The questions will take no more than 15 minutes.

We will take care to protect your privacy. None of the information we collect will identify you by name. Your participation is completely voluntary. You may refuse to answer any question that you do not want to answer. Your participation will not affect the care that your baby receives. If you would like to speak to someone from the Smart Hospital Project team, please visit our website https://www.smarthospitalproject.com.

We look forward to your involvement.

Brief identification:

() Mother () Father () Other: ___________

What was the gestational age of your baby at birth? ____weeks

For How long has your baby been in the NICU? __ weeks ___ days

Section 1: Your Experience with the Current Monitors

1. Your baby's vital signs (heart rate, oxygen level, breathing rate) are monitored using a machine that is connected to your baby using stickers and wires. How satisfied are you with the monitors?

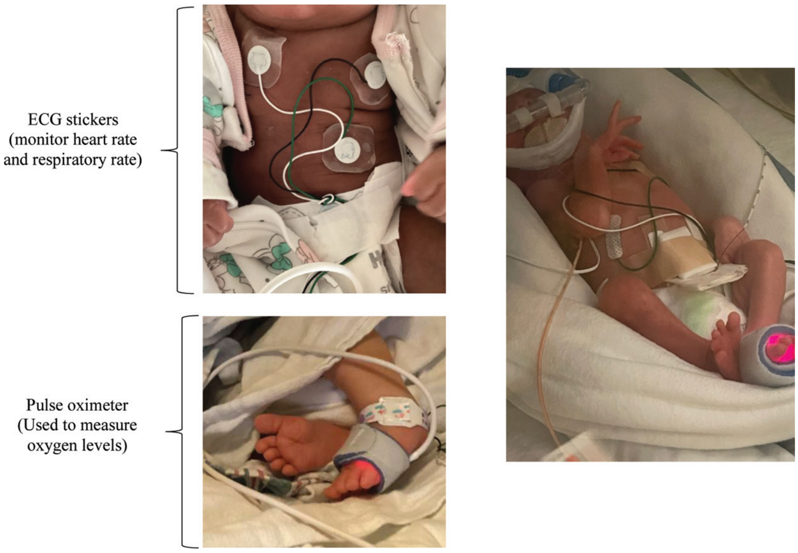

   a. Very dissatisfied
   b. Dissatisfied
   c. Neither satisfied nor dissatisfied
   d. Satisfied
   e. Very satisfied

2. What are your concerns with the monitors? (Mark all that apply—multiple choices are allowed)
   a. Monitor shows incorrect vital signs
   b. False alarms
   c. Problems with the stickers on my baby's skin
   d. Too many stickers on my baby's skin
   e. Too many wires around my baby
   f. Difficult to touch my baby because of the stickers and wires

 © 2025. The Author(s).

3. What kind of problems have you seen with the use of the current standard monitoring system? (Mark any that apply—multiple choices allowed)
   a. Skin lesions associated with ECG adhesives
   b. Skin lesions associated with the oxygen saturation probe.
   c. Pressure sore associated with ECG adhesives
   d. Pressure sore associated with the oximeter sensors.
   e. Parents afraid to handle their baby because of the multiple wires and cables
   f. Wires tangled around the baby's chest or limbs
   g. Wires soiled—requiring replacement or cleaning
   h. Cables soiled—requiring replacement or cleaning
   i. Sensors/wires disconnected
   j. Cables disconnected
   k. Wires broken or not working—requiring replacement
   l. Cables broken or not working—requiring replacement
4. The monitors and wires interfere with my ability to do skin-to-skin (kangaroo) care.
   a. Strongly disagree
   b. Disagree
   c. Neither agree nor disagree
   d. Agree
   e. Strongly agree
5. The stickers, wires, and monitors, prevent me from bonding with my baby.
   a. Strongly disagree
   b. Disagree
   c. Neither agree nor disagree
   d. Agree
   e. Strongly agree

Section 2: Wireless Monitoring in the NICU

6. What is your attitude toward new wireless technologies in the NICU?
   a. Very negative
   b. Negative
   c. Neutral
   d. Positive
   e. Very positive
7. I am worried about the safety and accuracy of the new wireless monitors.
   a. Strongly disagree
   b. Disagree
   c. Neither agree nor disagree
   d. Agree
   e. Strongly agree
8. What are your main concerns with the implementation of new wireless technology in the NICU? (Mark any that apply—multiple choices allowed)
   a. Accuracy (good and reliable data)
   b. Challenges using sensors
   c. Safety of stickers used to apply sensors
   d. Size of the sensors
   e. Weight of the sensor
   f. Battery life of wireless sensors
   g. Other (please specify):
9. Compared with the current monitors that use wires, what do you think is the cost of the wireless monitors?
   a. Less expensive
   b. The same cost
   c. More expensive
   d. No idea
   e. Other:

 © 2025. The Author(s).

10. What do you think might be the impact of a wireless system on your baby's hospital experience?
    a. Easier kangaroo care (KC) initiation
    b. Increase the amount of time on KC
    c. Reduce discomfort
    d. Decrease pain
    e. Better sleep
    f. Improve weight gain
    g. Earlier discharge home
    h. Other (please specify):

11. What do you think might be the impact of a wireless system on encouraging physical interaction between parents and their babies?
    a. Very negative
    b. Negative
    c. Neutral
    d. Positive
    e. Very positive

12. The radiation used with the wireless monitors is low and within regulated limits. Are you worried about the level of radiation?
    a. Not at all worried
    b. A little worried
    c. Very worried

13. Any additional comment (s)?

1B. Nurse and RT and PT Survey

Dear Sir/Madam,

The Montreal Children's Hospital has been funded by its Foundation to conduct a project to transform the way we care for our patients during hospitalization. One of the major pillars of the Smart Hospital Project is to test the use of new wireless technology to monitor vital signs such as heart rate, oxygen saturation, respiratory rate, and temperature in the NICU. The goal of this survey is to understand what is working well with the current system with wires and cables and to identify areas that need improvement. We also want to capture your thoughts concerning the use of wireless technology in the NICU environment.

As nurses provide very insightful information about our healthcare system, we are writing to request your participation in this study. With your help, we can meet the study goal of creating a better monitoring system for the patients/parents and healthcare professionals. If you agree to participate, we will ask you to complete a survey questionnaire, which will take approximately 15 minutes.

The information you provide will be kept confidential. It will not include your name or any other identifying information, and no one in the NICU will see your answers. If you would like more information about the research project, please speak with the Smart Hospital Project Team (*https://www.smarthospitalproject.com/*).

We look forward to your involvement.

ID: () Registered Nurse
() Neonatal Nurse Practitioner
Years of experience: () <2 years () 2–5 years () 6–10 years () >10 years

Section 1: Current State of Monitoring with Wires and Cables

1. How satisfied are you with the current standard monitoring system with wires and cables?
    a. Very dissatisfied
    b. Dissatisfied
    c. Neither satisfied nor dissatisfied

 © 2025. The Author(s).

d. Satisfied
e. Very Satisfied

2. What are your main concerns with the current standard monitoring system? (Mark any that apply—multiple choices allowed)
   a. Inaccurate readings
   b. False alarms
   c. Issues associated with ECG adhesives/oximeter heat generation/pressure of associated sensors.
   d. Too many sensors applied to the skin
   e. Too many wires around the baby
   f. Difficult to handle the baby because of the multiple wires and cables
3. The monitors and wires interfere with parents' ability to do skin-to-skin (kangaroo) care.
   a. Strongly disagree
   b. Disagree
   c. Neither agree nor disagree
   d. Agree
   e. Strongly agree
4. How often do you need to replace the sensors/wires during a regular 12-hour shift?
   a. Not at all
   b. Once
   c. Twice
   d. More than two times
   e. I don't know
   f. Other:
5. How often do you need to replace the cables during a regular 12-hour shift?
   a. Not at all
   b. Once
   c. Twice
   d. More than two times
   e. I don't know
   f. Other:
6. What kind of problems have you seen with the use of the current standard monitoring system? (Mark any that apply—multiple choices allowed)
   a. Skin lesions associated with ECG adhesives
   b. Skin lesions associated with the oximeter sensors.
   c. Pressure sore associated with ECG adhesives
   d. Pressure associated with the oximeter sensors.
   e. Parents afraid to handle their baby because of the multiple wires and cables
   f. Wires tangled around the baby's chest or limbs
   g. Wires soiled—requiring replacement or cleaning
   h. Cables soiled—requiring replacement or cleaning
   i. Wires disconnected
   j. Cables disconnected
   k. Wires broken or not working—requiring replacement
   l. Cables broken or not working—requiring replacement
   m. Other:
7. How much of your shift time do you estimate is needed to take care of the sensors/wires/cables?
   a. <5%
   b. 5–10%
   c. 11–20%
   d. 21–30%
   e. 31–40%
   f. >40%

 © 2025. The Author(s).

8. During a regular shift (12 hours) estimate the number of times you must attend to issues related to wires:

Number:

9. The stickers, wires, and monitors, prevent parents from bonding with their baby.
   a. Strongly disagree
   b. Disagree
   c. Neither agree nor disagree
   d. Agree
   e. Strongly agree

Section 2: Wireless Monitoring System in the NICU

10. What is your attitude toward new wireless technologies in the NICU?
   a. Very negative
   b. Negative
   c. Neutral
   d. Positive
   e. Very positive

11. I am worried about the safety and accuracy of the new wireless monitors.
   a. Strongly disagree
   b. Disagree
   c. Neither agree nor disagree
   d. Agree
   e. Strongly agree

12. What are your main concerns with the implementation of new wireless technology in the NICU? (Mark any that apply—multiple choices allowed)
   a. Accuracy (good and reliable data)
   b. Usability
   c. Safety
   d. Size of the sensors
   e. Weight of the sensor
   f. Battery life
   g. Other (please specify):

13. What do you think would be the cost of a wireless monitoring system in the NICU?
   a. Not expensive
   b. Less expensive than the actual wired system
   c. More expensive than the actual wired system
   d. No idea if more or less
   e. Other:

14. What do you think might be the impact of a wireless system on encouraging physical interaction between parents and their babies?
   a. Very negative
   b. Negative
   c. Neutral
   d. Positive
   e. Very positive

15. What do you think might be the impact of a wireless system on outcomes?
   a. Improve kangaroo care (KC) experience
   b. Increase the amount of time on KC
   c. Reduce discomfort
   d. Decrease pain
   e. Better sleep
   f. Improve weight gain

 © 2025. The Author(s).

g. Earlier discharge home
h. Other (please specify):

16. The radiation used with the wireless monitors is low and within regulated limits. Are you worried about the level of radiation?
   a. Not at all worried
   b. A little worried
   c. Very worried

17. Any additional comment (s)?

1C. Physician Survey

Dear Sir/Madam,

The Montreal Children's Hospital has been funded by its foundation to conduct a project to transform the way we care for our patients during hospitalization. One of the major pillars of the Smart Hospital Project is to test the use of new wireless technology to monitor vital signs such as heart rate, oxygen saturation, respiratory rate, and temperature in the NICU. The goal of this survey is to understand what is working well or not with the current system with wires and cables and to identify areas that need improvement. Also, to capture your thoughts concerning the use of wireless technology in the NICU environment.

As physicians always provide very insightful information about our health care system we are writing to request your participation in this study. With your help we can meet the study goals, creating a better monitoring system for the patients/parents and HCP. If you agree to participate, we will ask you to complete a survey questionnaire (which will take ~15 minutes). Most of it can be filled out quickly.

The information you provide will be kept confidential. It will not include your name or any other identifying information, and no one in the NICU will see your answers. If you would like more information about the research, please speak with the Smart Hospital Project Team (https://www.smarthospitalproject.com/).

We look forward to your involvement.

Brief identification:

() Neonatologist () Neonatal Fellow () Pediatric Resident () Other: ___________

Years of experience: () <2 years () 2–5 years () 6–10 years () >10 years

Section 1: Current State of Monitoring with Wires and Cables

1. How satisfied are you with the current standard monitoring system with wires and cables?
   a. Very dissatisfied
   b. Dissatisfied
   c. Neither satisfied nor dissatisfied
   d. Satisfied
   e. Very satisfied

2. What are your main concerns with the current standard monitoring system? (Mark any that apply—multiple choices allowed)
   a. Inaccurate readings
   b. False alarms
   c. Issues associated with adhesion of the sensors (heat generation or pressure and skin irritation)
   d. Too many sensors applied to the skin
   e. Too many wires around the baby
   f. Difficult to handle the baby because of the multiple wires and cables

3. What kind of problems have you seen with the use of the current standard monitoring system? (Mark any that apply—multiple choices allowed)
   a. Skin lesions associated with ECG adhesives
   b. Skin lesions associated with the oxygen saturation sensor.
   c. Pressure sores associated with ECG adhesives
   d. Pressure sores associated with the oximeter sensors.
   e. Parents afraid to handle their baby because of the multiple wires and cables
   f. Wires tangled around the baby's chest or limbs
   g. Wires soiled—requiring replacement or cleaning
   h. Cables soiled—requiring replacement or cleaning
   i. Wires disconnected
   j. Cables disconnected
   k. Wires broken or not working—requiring replacement

 © 2025. The Author(s).

l. Cables broken or not working—requiring replacement
m. Other:

4. The monitors and wires interfere with parents' ability to do skin-to-skin (kangaroo) care.
   a. Strongly disagree
   b. Disagree
   c. Neither agree nor disagree
   d. Agree
   e. Strongly agree

5. The stickers, wires, and monitors, prevent parents from bonding with their baby.
   a. Strongly disagree
   b. Disagree
   c. Neither agree nor disagree
   d. Agree
   e. Strongly agree

6. What proportion of time in a nurse's shift do you estimate is needed to take care of the sensors/wires/cables?
   a. <5%
   b. 5–10%
   c. 11–20%
   d. 21–30%
   e. 31–40%
   f. >40%

7. How often do you think the sensors/wires need to be replaced during a regular 12-hour shift?
   a. Never
   b. Once
   c. Twice
   d. More than two times
   e. I don't know
   f. Other:

8. During a regular shift (12 hours) what's the number of times you think a nurse must take care of issues related to wires:

Number:

9. How often do you need to replace the cables during a regular 12-hour shift?
   a. Never
   b. Once
   c. Twice
   d. More than two times
   e. I don't know
   f. Other:

Section 2: Wireless Monitoring System in the NICU

10. What is your attitude toward new wireless technologies in the NICU?
   a. Strongly oppose
   b. Somewhat oppose
   c. Neither favor nor oppose
   d. Somewhat favor
   e. Strongly favor

11. I am worried about the safety and accuracy of the new wireless monitors.
   a. Strongly disagree
   b. Disagree
   c. Neither agree nor disagree
   d. Agree
   e. Strongly agree

12. What are your main concerns with the implementation of new wireless technology in the NICU? (Mark any that apply—multiple choices allowed)
    a. Accuracy (good and reliable data)
    b. Challenges related to "user-friendliness" of technology
    c. Safety
    d. Size of the sensors
    e. Weight of the sensor
    f. Battery life
    g. Other (please specify):
13. What do you think would be the cost of a wireless monitoring system in the NICU?
    a. Not expensive
    b. Less expensive than the actual wired system
    c. More expensive than the actual wired system
    d. No idea
    e. Other:
14. What do you think might be the impact of a wireless system on encouraging physical interaction between parents and their babies?
    a. Very negative
    b. Negative
    c. Neither negative nor positive
    d. Positive
    e. Very positive
15. What do you think might be the impact of a wireless system on outcomes?
    a. Improve kangaroo care (KC) experience
    b. Increase the amount of time on KC
    c. Reduce discomfort
    d. Decrease pain
    e. Better sleep
    f. Improve weight gain
    g. Earlier discharge home
    h. Other (please specify):
16. The radiation used with the wireless monitors is low and within regulated limits. Are you worried about the level of radiation?
    a. Not at all worried
    b. A little worried
    c. Very worried
17. Any additional comment(s)?

Supplementary Material S2 CHERRIES checklist—checklist for reporting results of internet e-surveys (CHERRIES)

Item category	Checklist item	Explanation (expands on information provided in manuscript)	Page number (if directly mentioned in the manuscript)
Design	Describe survey design	Survey designed with input from neonatologists, nurses, and parents. Circulated using links provided by email and social media platforms	5–7
IRB (Institutional Review Board) approval and informed consent process	IRB approval	Exemption for informed consent was obtained from the Institution Research Ethics Board, as the responses and their aggregate analysis were considered secondary use of anonymous information (Tri council policy statement 2022, Article 2.4)	5

(*Continued*)

Item category	Checklist item	Explanation (expands on information provided in manuscript)	Page number (if directly mentioned in the manuscript)
	Informed consent	The subjects were informed of the purpose of the survey, anonymity, confidentiality, and voluntary principles on the first page of the survey prior to proceeding with questions	5
	Data protection	The questionnaire did not ask for any identifying information and did not collect any identifying information (i.e., IP address)	5
Development and pretesting	Development and testing	Initial surveys were developed and circulated as Word documents to neonatologists, nurses, and parents who provided feedback for improving clarity	5
Recruitment process and description of the sample having access to the questionnaire	Open survey versus closed survey	Open survey. Anyone can access the provided survey link	7
	Contact mode	Contact was made by circulating links via email and posting links on social media platforms of PI's, including in relevant Facebook professional groups	6–7
	Advertising the survey	We promoted the survey by leveraging the professional networks of the PIs, sending links to colleagues, and sharing on social media platforms	6–7
Survey administration	Web/e-mail	Survey links were circulated by emailing links which led to the LimeSurvey online platform where the survey was completed	5–7
	Context	The commercial platform specializes in online questionnaires. We only used it to collect survey responses	5–7
		Mandatory/voluntary	The survey was voluntary
6			
	Incentives	No incentives were provided	7
	Time/date	This data was collected between March and July 2023	5
	Randomization of items or questionnaires	Question order was not randomized	5
	Adaptive questioning	NA	NA
	Number of Items	Survey had 20 question items (3 related to the respondent professional title/years of experience, 17 questions on the survey topic)	5
	Number of screens (pages)	3 pages	7
	Completeness check	NA	NA
	Review step	Respondents were able to move forward and backward freely in the survey	7
Response rates	Unique site visitor	Survey system did not record the unique site of visitors	NA
	View rate (Ratio of unique survey		NA

(*Continued*)

(*Continued*)

Item category	Checklist item	Explanation (expands on information provided in manuscript)	Page number (if directly mentioned in the manuscript)
	visitors/unique site visitors)	The survey is voluntary. The system cannot record the number of unique visitors, so the view rate cannot be calculated	
	Participation rate (Ratio of unique visitors who agreed to participate/unique first survey page visitors)	This information was not recorded	NA
	Completion rate (Ratio of users who finished the survey/users who agreed to participate)	This was a voluntary questionnaire. Therefore, completion of the survey implied consent to participate, and thus we cannot consider people who intended to respond but did not	NA
	Cookies used	Cookies were not used	NA
Preventing multiple entries from the same individual	IP check	Did not check participant IP	7
	Log file analysis	NA	NA
	Registration	NA	NA
	Handling of incomplete questionnaires	Incomplete surveys were included as long as basic questions about the participant's professional title were completed to try and ensure the appropriateness of respondents	7
Analysis	Questionnaires submitted with an atypical timestamp	Information about the time taken to complete the survey was collected but no cut-offs were applied	7
	Statistical correction	No weighting or propensity score corrections were applied. Only nonparametric statistical methods were utilized	7–8

 © 2025. The Author(s).

Supplementary Table S1 Correlational analysis of Likert survey responses

Variable 1	Variable 2	Data conversion	Correlation coefficient (*r*)
Length of stay	Q1. Satisfaction with the current monitoring system	[1 = very dissatisfied, 5 = very satisfied]	$r = 0.03$
	Q4. Interference with skin-to-skin care	[1 = strongly disagree 5 = strongly agree]	$r = 0.22$
	Q5. Interference with parent–infant bonding	[1 = strongly disagree 5 = strongly agree]	$r = 0.22$
	Q10. Attitude toward wireless monitoring system	[1 = very negative, 5 = very positive]	$r = 0.17$
	Q11. Concerns about safety and accuracy	[1 = strongly disagree 5 = strongly agree]	$r = -0.24$
	Q15. Impact of wireless system on physical interaction	[1 = very negative, 5 = very positive]	$r = 0.24$
	Q16. Radiation concerns	[1= not at all worried, 3 = very worried]	$r = 0.15$
GA of baby	Q1. Satisfaction with the current monitoring system	[1 = very dissatisfied, 5 = very satisfied]	$r = -0.43$
	Q4. Interference with skin-to-skin care	[1 = strongly disagree 5 = strongly agree]	$r = 0.33$
	Q5. Interference with parent–infant bonding	[1 = strongly disagree 5 = strongly agree]	$r = 0.04$
	Q10. Attitude toward wireless monitoring system	[1 = very negative, 5 = very positive]	$r = 0.44$
	Q11. Concerns about safety and accuracy	[1 = strongly disagree 5 = strongly agree]	$r = 0.18$
	Q15. Impact of wireless system on physical interaction	[1 = very negative, 5 = very positive]	$r = 0.25$
	Q16. Radiation concerns	[1 = not at all worried, 3 = very worried]	$r = -0.15$

Supplementary Table S2 Perspective—current wired monitoring system

Survey item	Parents	Physicians	Nurse, PT, RT
Satisfaction	$n = 25$	$n = 376$	$n = 588$
Very dissatisfied	1 (4) [0.7–20]	31 (8) [6–11]	63 (11) [8–14]
Dissatisfied	2 (8) [2–25]	68 (18) [15–22]	137 (23) [20–27]
Neither Satisfied nor Dissatisfied	9 (36) [20–55]	150 (40) [35–45]	232 (39) [36–45]
Satisfied	11 (44) [26–63]	121 (32) [28–37]	144 (24) [21–28]
Very Satisfied	2 (8) [2–25]	6 (2) [0.7–4]	12 (2) [1–4]
Interference with skin-to-skin care	$n = 24$	$n = 376$	$n = 588$
Strongly disagree	2 (8) [2–26]	8 (2) [1–4]	13 (2) [1–4]
Disagree	2 (8) [2–26]	35 (9) [7–13]	87 (15) [12–18]
Neither agree nor disagree	6 (25) [12–45]	51 (14) [11–18]	122 (21) [18–24]
Agree	8 (33) [18–53]	218 (58) [53–63]	267 (45) [41–50]
Strongly agree	6 (25) [12–45]	64 (17) [14–21]	99 (17) [14–20]

Note: Results are presented as *n* (%; 95% confidence interval of the %).

 © 2025. The Author(s).

Supplementary Table S3 Perspective—future wired monitoring system

Survey item	Parents	Physicians	Nurse, PT, RT
Attitude toward wireless technology	$n = 25$	$n = 357$	$n = 568$
Very negative	0 (0) [0.0–13]	0 (0) [0.0–1]	3 (0.5) [0.0–2]
Negative	1 (4) [0.7–20]	2 (0.6) [0–2]	2 (0.4) [0.0–1]
Neutral	9 (36) [20–55]	31 (9) [6–12]	57 (10) [8–13]
Positive	7 (28) [14–48]	90 (25) [21–30]	175 (31) [27–35]
Very positive	8 (32) [17–52]	234 (66) [60–70]	318 (56) [52–60]
Impact on the physical interaction between parents and infants	$n = 25$	$n = 357$	$n = 568$
Very negative	0 (0) [0 0.0–13]	0 (0) [0.0–1]	0 (0) [0.0–0.7]
Negative	0 (0) [0.0–13]	0 (0) [0.0–1]	3 (0.5) [0.2–1.6]
Neutral	1 (4) [0.7–20]	20 (6) [4–8]	28 (5) [3–7]
Positive	14 (56) [37–73]	106 (30) [25–34]	177 (31) [27–35]
Very positive	10 (40) [23–59]	231 (65) [60–69]	360 (63) [59.3–67.3]

Note: Results are presented as *n* (%; 95% confidence interval of the %).

Supplementary Table S4 Statistical test for intergroup associations between years of practice and physician responses

Question	*p*-Value	Secondary test responses grouped	*p*-Value
Q1. Satisfaction with the current monitoring system	0.3612	Satisfied—Neutral—Dissatisfied	0.2441
Q4. Interference with skin-to-skin care	0.1833	Agree—Neutral—Disagree	0.0343
Q5. Interference with parent–infant bonding	0.4547	Agree—Neutral—Disagree	0.8873
Q6. Proportion of nurse's time spent dealing with sensors/wires/cables	0.4178	<20 to 21–40 to >40%	0.2018
Q7. How often are sensors/wires replaced during the 12-h shift	0.5880	"Other" and "I don't know" removed	0.6829
Q9. Number of times during shift must address wires	0.1913	"Other" and "I don't know" removed	0.7064
Q10. Attitude toward wireless monitoring system	0.2728	Positive—Neutral—Negative	0.3765
Q11. Concerns about safety and accuracy	0.3002	Agree—Neutral—Disagree	0.2598
Q13. Cost perception	0.1699	Remove "other" responses	0.0732
Q15. Impact of wireless system on physical interaction	0.4532	–	–
Q16. Radiation concerns	0.7477	–	–

 © 2025. The Author(s).

Supplementary Table S5 Statistical test for intergroup associations in nurse, PT, and RT survey responses

Variable 1	Variable 2	Test	Reject null (test)	*p*-Value	Secondary test	Reject null (test)	*p*-Value
Years of Practice	Q1. Satisfaction with the current monitoring system	Chi-square ind	No	0.1041	Group responses into satisfied, neutral, dissatisfied	No	0.0516
	Q4. Interference with skin-to-skin care	Chi-square ind	No	0.5971	Grouped into agree, neutral, disagree	No	0.9745
	Q5. Interference with parent–infant bonding	Chi-square ind	No	0.5180	Grouped into agree, neutral, disagree	No	0.7008
	Q6. Proportion of nurses' time spent dealing with sensors/wires/cables	Chi-square ind	No	0.3033	Grouped into <5–20%, 21–40%, >40%	No	0.1343
	Q7. How often are sensors/wires replaced during 12-h shift	Chi-square ind	No	0.7324	Remove "other" and "I don't know"	No	0.8842
	Q9. Number of times during shift must address wires	Chi-square ind	No	0.7306	NA	No	0.4334
	Q10. Attitude toward wireless monitoring system	Chi-square ind	No	0.5656	Grouped into pos, neutral, and neg	No	0.2064
	Q11. Concerns about safety and accuracy	Chi-square ind	No	0.5883	Grouped into agree, neutral, disagree	No	0.2787
	Q13. Cost perception	Chi-square ind	No	0.6617	Remove "other" responses	No	0.6920
	Q15. Impact of wireless system on physical interaction	Chi-square ind	No	0.2656	NA	NA	NA
	Q16. Radiation concerns	Chi-square ind	No	0.2691	NA	NA	NA

 © 2025. The Author(s).

Supplementary Table S6 Chi-square test of independence to assess for differences between groups 2 and 3 responses

Type of HCP (group 2 versus 3)	*p*-Value	Secondary test	*p*-Value
Q1. Satisfaction with the current monitoring system	0.0468	Chunked into satisfied, neutral, or dissatisfied	0.0144
Q4. Interference with skin-to-skin care	<0.0001	Chunked into agree, neutral, disagree	0.0002
Q5. Interference with parent–infant bonding	0.1389	Chunked into agree, neutral, disagree	0.0336
Q6. Proportion of nurses' time spent dealing with sensors/wires/cables	<0.0001	Grouped into <5–20%, 21–40%, >40%	<0.0001
Q7. How often are sensors/wires replaced during 12-h shift	<0.0001	Remove "other" and "I don't know responses"	<0.0001
Q9. Number of times during shift must address wires	<0.0001	Remove "other" and "I don't know responses"	<0.0001
Q10. Attitude toward wireless monitoring system	0.0871	Chunked into pos, neutral, and neg	0.6109
Q11. Concerns about safety and accuracy	0.8641	Chunked into agree, neutral, disagree	0.7341
Q13. Cost perception	0.0747	Remove the "other" option	0.0732
Q15. Impact of wireless system on physical interaction	0.5141	Chunked into pos, neutral, and neg	0.3540
Q16. Radiation concerns	0.360	NA	NA

 © 2025. The Author(s).
